# Characteristics, outcomes, and predictors of *de novo* malignancy after heart transplantation

**DOI:** 10.3389/fcvm.2022.939275

**Published:** 2022-08-08

**Authors:** Jong-Chan Youn, Darae Kim, In-Cheol Kim, Hye Sun Lee, Jin-Oh Choi, Eun-Seok Jeon, Keith Nishihara, Evan P. Kransdorf, David H. Chang, Michelle M. Kittleson, Jignesh K. Patel, Danny Ramzy, Fardad Esmailian, Jon A. Kobashigawa

**Affiliations:** ^1^Department of Cardiology, Cedars-Sinai Medical Center, Smidt Heart Institute, Los Angeles, CA, United States; ^2^Division of Cardiology, Department of Internal Medicine, Catholic Research Institute for Intractable Cardiovascular Disease, College of Medicine, Seoul St. Mary's Hospital, The Catholic University of Korea, Seoul, South Korea; ^3^Division of Cardiology, Department of Medicine, Samsung Medical Center, Heart Vascular Stroke Institute, Sungkyunkwan University School of Medicine, Seoul, South Korea; ^4^Division of Cardiology, Department of Internal Medicine, Keimyung University Dongsan Hospital, Daegu, South Korea; ^5^Biostatistics Collaboration Unit, Yonsei University College of Medicine, Seoul, South Korea; ^6^Department of Cardiothoracic Surgery, Cedars-Sinai Medical Center, Smidt Heart Institute, Los Angeles, CA, United States

**Keywords:** post-transplant malignancy, prognosis, heart transplant, de novo malignancies after heart transplantation, outcome

## Abstract

**Background:**

Post-transplant malignancy (PTM) causes long-term morbidity and mortality in heart transplant (HTx) recipients. However, the detailed characteristics or predictors of PTM are not well-known. We evaluated the incidence, characteristics, long-term outcomes, and predictors of *de novo* PTM using a single center large-volume database.

**Methods:**

We retrospectively analyzed the types and characteristics of *de novo* PTM in 989 patients who underwent HTx. Univariate and multivariate logistic regression analyses were used for the PTM prediction model.

**Results:**

Two hundred and six patients (20.8%) had *de novo* PTMs (241 cancers) during a median follow-up of 11.5 years. PTM patients were older than non-PTM patients, received immunosuppressive therapy for a longer period, and were more likely to be male and white. Skin cancers were the most frequent types of malignancy (60.6%) followed by prostate (9.5%), lung (7.1%), and breast (4.1%) cancers. Although most cancers (88.8%) were surgically resected at initial presentation, about half (47.3%) recurred or progressed. Patients with skin cancer and non-skin cancer had significantly lower overall survival (*P* < 0.001) than patients without cancer. Older age (*P* < 0.001), white race (*P* = 0.001), and longer time receiving immunosuppressive therapy (*P* < 0.001) were independent predictors for PTM.

**Conclusion:**

Older age, white race, and longer administration of immunosuppressive therapies were independent risk factors for PTM, which was associated with increased mortality. Further research is necessary for the prevention and early detection of PTM in HTx recipients.

## Introduction

Heart transplantation (HTx) is the optimal treatment for selected patients with end-stage heart failure (1). However, post-transplant malignancy (PTM) has been a significant cause of long-term morbidity and mortality in HTx recipients (2–7). According to the recent worldwide International Society for Heart and Lung Transplantation (ISHLT) registry data, PTM continues to be a significant cause of long-term morbidity, with an incidence of 28% at 10 years post-transplant (8, 9). Previously, we reported that >10% of adult HTx recipients developed de novo PTM between 1 and 5 years after transplantation, which was associated with increased mortality (2). However, we could not verify detailed cancer-related information or the recipients' ethnicities due to limitations of the ISHLT registry data. Therefore, we sought to evaluate the incidence, characteristics, long-term outcomes, and predictors of *de novo* PTM more comprehensively using a large-volume, detailed HTx database.

## Methods

### Study population

We retrospectively analyzed the types and characteristics of *de novo* PTM in a consecutively enrolled cohort of 1,062 patients who underwent HTx between January 1997 and December 2013 from a single center. Seventy-three patients had a history of pre-transplant malignancy and were excluded from this study. All HTx patients had cancer surveillance according to American Cancer Society guidelines. They had close skin cancer surveillances, including educations on preventive measures, and yearly dermatologic examinations and were screened for breast, colon, and prostate cancer in the same manner as the general population. Among 989 consecutively enrolled patients without a pre-transplant history of malignancy, 206 patients developed *de novo* PTMs (241 cancers) during a median follow-up period of 11.5 years. We investigated the incidence, clinical characteristics, outcomes, and predictors of PTM during the same study period. The Cedars-Sinai Institutional Review Board approved the study. Written informed consent was obtained from all enrolled patients.

### Clinical outcomes

Post-transplant clinical outcomes included overall survival, cause of death, 10-year freedom from angiographic cardiac allograft vasculopathy (CAV, defined as any stenosis ≥30%), non-fatal major adverse cardiac events (NF-MACE, defined as the development of myocardial infarction, new congestive heart failure, need for percutaneous coronary intervention/angioplasty, placement of a pacemaker or implantable cardioverter-defibrillator, and stroke), any treated rejection (ATR), acute cellular rejection (ACR, t defined as biopsy proven grade 2 or 3 cellular rejection), and antibody-mediated rejection (AMR, defined as biopsy proven grade 2 or 3 antibody-mediated rejection). Rejections were diagnosed according to the revised International Society for Heart and Lung Transplantation (ISHLT) classification (10, 11).

### Immunosuppressive therapy

Induction therapy with anti-thymocyte globulin (ATG) was indicated for sensitized patients with panel reactive antibodies >10%, patients with renal insufficiency (creatinine > 2 mg/dL), and patients receiving multiple organ transplants. Induction dose with ATG was a 1.5 mg/kg daily dose for 5 days post HTx. Calcineurin inhibitor (CNI)-based triple immunosuppressive therapy (tacrolimus, mycophenolate mofetil, and prednisone) was administered initially as maintenance therapy to most patients. Cyclosporine was administered if patients developed severe side effects from tacrolimus, such as seizures or encephalopathy. A regimen using a mammalian target of rapamycin (mTOR) inhibitor, either sirolimus or everolimus, in place of a CNI-free regimen was prescribed for eligible patients, including those with renal insufficiency or PTM. An mTOR inhibitor was administered in conjunction with a CNI in patients who developed rejection with graft dysfunction, cytomegalovirus (CMV) infection, or CAV. In case of intolerance to an mTOR inhibitor, a conventional CNI-based regimen was maintained. Patients at low risk for rejection were weaned off steroids 6 months after HTx (12).

### Statistical analysis

Categorical variables are summarized as frequencies and percentages of the total group. Continuous variables are summarized as mean ± standard deviation. Discrete variables were compared using chi-square test, and continuous variables using Student's *t*-test or Mann-Whitney *U*-test. The cumulative incidence of events was assessed using the Kaplan-Meier method, and the statistical significance was calculated using the log-rank test. To avoid immortal time bias, all cumulative Kaplan–Meier estimates of clinical outcomes, including survival and morbidity (CAV, NF-MACE, ATR, ACR, and AMR), were assessed using landmark analysis. The median time from HTx to the first *de novo* malignancy (6.4 years) was used as a landmark time point. However, the number of clinical events, including mortality (number of deaths and cause of deaths) and morbidity (CAV, NF-MACE, ATR, ACR, and AMR) outcomes, was analyzed for the entire study period. Univariate and multivariate logistic regression analyses were used for the PTM prediction model. We established a cubic spline curve to better understand the relationship of the probability of cancer development with recipient age and time receiving immunosuppressive therapy. For the probability curves of cancer by recipient age, time receiving immunosuppressive therapy, white race, male sex, ischemic time, and pre-transplant mechanical circulatory support were adjusted. A *P* < 0.05 was considered statistically significant. Statistical analyses were performed using SPSS version 20.0 (IBM Corporation, Armonk, NY, USA) or R Statistical Package (Institute for Statistics and Mathematics, Vienna, Austria, ver. 4.0.0, www.R-project.org).

## Results

### Baseline characteristics of the study population

Among 989 consecutively enrolled patients without a pre-transplant history of malignancy who underwent HTx between 1997 and 2013, 206 (20.8%) developed *de novo* PTMs (241 cancers) during a median follow-up period of 11.5 years (interquartile range 7.5–14.8 years). Baseline clinical characteristics of the study population at the time of transplantation are shown in Table 1. Compared to patients without PTM, patients with PTM tended to be older and receive immunosuppressive therapy longer and were more likely to be male and white. Among patients with PTM, 77 (37.4%) patients had a first degree family history of cancer.

**Table 1:** Baseline characteristics of patients with and without post-transplant malignancy.

**Variables**	**PTM (*n* = 206)**	**No PTM (*n* = 783)**	***P***
Recipient age (years)	60.8 ± 9.3	53.5 ± 12.8	<0.001
Recipient gender (male, %)	169 (82.0%)	578 (73.8%)	0.014
Recipient race			<0.001
Caucasian/White	179 (86.9%)	539 (68.8%)	
Black	10 (4.9%)	107 (13.7%)	
Latino/Hispanic	7 (3.4%)	71 (9.1%)	
Asian/Pacific Islander	10 (4.9%)	66 (8.4%)	
Reasons for transplant (%)			0.104
Ischemic	114 (55.3%)	339 (43.3%)	
Idiopathic	62 (30.1%)	298 (38.1%)	
Congenital	19 (9.2%)	66 (8.4%)	
Amyloid	5 (2.4%)	19 (2.4%)	
Sarcoid	1 (0.5%)	4 (0.5%)	
Others	5 (2.4%)	55 (7.0%)	
BMI (kg/m^2^)	25.4 ± 4.3	25.0 ± 4.4	0.188
Hypertension (%)	67 (32.5%)	304 (38.8%)	0.062
Diabetes (%)	51 (24.8%)	185 (23.6%)	0.991
Pre-transplant MCS (%)	20 (9.7%)	153 (19.5%)	0.001
Multi-organ transplant (%)	9 (4.4%)	54 (6.9%)	0.202
Pregnancy history (%)	27 (13.1%)	154 (19.7%)	0.692
Donor age (years)	32.2 ± 12.6	32.9 ± 12.6	0.496
Induction therapy with ATG (%)	69 (33.5%)	296 (37.8%)	0.838
High-risk CMV mismatch (%)	46 (22.3%)	164 (20.9%)	0.394
Total ischemic time (minutes)	197.8 ± 59.0	186.4 ± 63.4	0.024
Length of hospital stay (days)	15.1 ± 13.3	16.1 ± 16.1	0.447
Length of ICU stay (days)	8.0 ± 6.9	7.6 ± 7.0	0.598
Time on immunosuppressive therapy (years)	11.4 ± 4.8	8.7 ± 5.3	<0.001

Values are mean ± standard deviation or number (%). Categorical variables were compared using the chi-squared method, and independent t-tests were used for the continuous variables.

PTM, post-transplant malignancy; BMI, body mass index; MCS, mechanical circulatory support; ATG, anti-thymocyte globulin; CMV, cytomegalovirus; ICU, intensive care unit.

### Types, frequencies, and characteristics of post-transplant malignancies

Detailed types, frequencies, and characteristics of *de novo* PTMs (n = 241 cancers) in 206 patients are shown in Table 2. The median time from HTx to the first malignancy was 6.4 years (interquartile range 3.1–9.8 years). Non-melanoma skin cancers were the most frequent types of malignancy (57.3%), followed by prostate cancers (9.5%), lung cancers (7.1%), and breast cancers (4.1%). Seventy-seven patients (37.4%) had a positive family history of cancer in first-degree relatives. At the time of initial diagnosis of PTM, 102 cancers (42.3%) had multiple or extensive disease statuses. While most cancers (88.8%) were surgically resected at initial presentation, about half (47.3%) recurrence or progressed. At the time of initial presentation, all skin cancers were surgically resected, but there was a relatively high recurrence rate (48.3%). Melanoma was the most likely to have multiple or extensive disease statuses (62.5%) at initial presentation, while lung cancer had the highest rate of recurrence or disease progression (76.5%).

**Table 2:** Types, frequencies, and characteristics of post-transplant de novo malignancies.

**Types of cancer**	**1st cancer**	**2nd cancer**	**3rd cancer**	**All cancers**	**Multiple or extensive disease**	**Surgical resection**	**Recurrence or disease progression**
Non-melanoma skin cancer							
Squamous cell carcinoma	105	14	1	120 (49.8%)	59 (49.2%)	120 (100%)	59 (49.2%)
Basal cell carcinoma	13	3	–	16 (6.6%)	8 (50.0%)	16 (100%)	5 (31.3%)
Merkel cell carcinoma	2	–	–	2 (0.8%)	1 (50.0%)	2 (100%)	1 (50.0%)
Melanoma	7	1	–	8 (3.3%)	5 (62.5%)	8 (100%)	4 (50.0%)
Prostate carcinoma	17	3	3	23 (9.5%)	3 (13.0%)	14 (60.9%)	7 (30.4%)
Lung carcinoma	10	7	–	17 (7.1%)	10 (58.8%)	12 (70.6%)	13 (76.‘5%)
Breast carcinoma	9	1	–	10 (4.1%)	3 (30.0%)	8 (80.0%)	4 (40.0%)
Head & neck carcinoma	7	–	–	7 (2.9%)	1 (14.3%)	5 (71.4%)	2 (28.6%)
Colorectal carcinoma	7	–	–	7 (2.9%)	2 (28.6%)	7 (100%)	5 (71.4%)
Lymphoproliferative disorder	7	–	–	7 (2.9%)	4 (57.1%)	2 (28.6%)	5 (71.4%)
Bladder carcinoma	5	2	–	7 (2.9%)	1 (14.3%)	7 (100%)	2 (28.6%)
Renal cell carcinoma	4	–	–	4 (1.7%)	0 (0%)	4 (100%)	1 (25.0%)
Esophageal carcinoma	2	–	–	2 (0.8%)	1 (50.0%)	1 (50.0%)	1 (50.0%)
Vulvar carcinoma	2	–	–	2 (0.8%)	0 (0%)	2 (100%)	0 (0%)
Others*	9	–	–	9 (3.7%)	4 (44.4%)	6 (66.7%)	5 (55.6%)
Skin cancers	127	18	1	146 (60.6%)	73 (50.0%)	146 (100%)	69 (47.3%)
Non-skin cancers	79	13	3	95 (39.4%)	29 (30.5%)	68 (71.6%)	45 (47.4%)
Total	206	31	4	241 (100%)	102 (42.3%)	214 (88.8%)	114 (47.3%)

^*^Brain, thyroid, stomach, liver, neuroendocrine, pancreas, cervix, urethra, and leukemi.

### Clinical outcomes of patients with post-transplant malignancies

When patients with PTM were divided into patients with skin cancer and those with non-skin cancer, both groups had significantly higher mortality than the control group during the entire study period (42.2 and 65.6%, respectively, vs. 39.0%, *P* < 0.001; Table 3). When patients with skin cancer were further classified into non-melanoma skin cancer vs. melanoma groups, mortality rates were similar between non-melanoma skin cancer group and the control group (41.7 vs. 39.0%, *p* = 0.714; Supplementary Table 1). For patients without PTM, the most common causes of death were cardiac-related (43.3%), including rejection and CAV, followed by infection (21.6%). For patients with PTM, the most com patients with non-melanoma skin cancer showed comparable morality with non-PTM group mon causes of death were malignancy (total: 44.4%; melanoma: 100%, non-melanoma skin cancer: 24.4% and non-skin cancer: 55.9%), followed by infection (total: 19.4%; melanoma: 0%, non-melanoma skin cancer: 17.8% and non-skin cancer: 25.4%) and cardiac-related (total: 10.2%; melanoma: 0%, non-melanoma skin cancer: 13.3%, and non-skin cancer: 5.1%) (Supplementary Table 1). When the median time from HTx to the first *de novo* malignancy (6.4 years) was used as a landmark time point, both patients with skin cancer and patients with non-skin cancer had a significantly lower overall survival (59.6 and 41.7%, respectively, vs. 78.1%, *P* < 0.001) than those without PTM. Regarding the morbidity outcomes, patients with skin cancers had a significantly increased incidence of CAV (39.7 vs. 23.4%, *P* < 0.001) and a lower 10-year freedom from CAV (65.4% vs. 75.3%, *P* = 0.02) than patients without PTM. There were no significant differences between patients with skin cancer and non-skin cancer in terms of morbidity or mortality outcomes (Table 3).

**Table 3:** Clinical outcome of patients with and without post-transplant malignancy.

**Variables**	**Skin cancer (*n* = 116)**	**Non-skin cancer (*n* = 90)**	**No cancer (*n* = 783)**	***P***
**Mortality outcomes**
Deaths	49 (42.2%)*	59 (65.6%)*	305 (39.0%)	<0.001
Cause of death				<0.001
Cardiac	8 (16.3%)	3 (5.1%)	132 (43.3%)	
Infection	6 (12.2%)	15 (25.4%)	66 (21.6%)	
Malignancy	15 (30.6%)	33 (55.9%)	0 (0%)	
Renal	3 (6.1%)	1 (1.7%)	14 (4.6%)	
Cerebrovascular	0 (0%)	2 (3.4%)	10 (3.3%)	
Others	17 (34.7%)	5 (8.5%)	83 (27.2%)	
Overall survival	59.6%*	41.7%*	78.0%	<0.001
**Morbidity outcomes**
CAV	46 (39.7%)*	26 (28.9%)	183 (23.4%)	0.001
NF-MACE	37 (31.9%)	30 (33.3%)	199 (25.4%)	0.120
ATR	23 (19.8%)	13 (14.4%)	145 (18.5%)	0.577
ACR	13 (11.2%)	6 (6.7%)	74 (9.5%)	0.539
AMR	6 (5.2%)	8 (8.9%)	56 (7.2%)	0.578
10-year freedom from CAV	65.4%*	68.1%	75.3%	0.040
10-year freedom from NF-MACE	73.1%	69.4%	76.7%	0.341
10-year freedom from ATR	80.8%	86.1%	80.9%	0.509
10-year freedom from ACR	88.5%	94.4%	90.6%	0.396
10-year freedom from AMR	94.2%	93.1%	92.4%	0.796

To avoid immortal time bias, all cumulative Kaplan-Meier estimates of clinical outcomes were assessed using landmark analysis. The median time from heart transplant to first de novo malignancy (6.4 years) was used as the landmark time point. However, the numbers of clinical events, including mortality and morbidity outcomes, were analyzed for the entire study period.

^*^Significant with a P < 0.05 for skin cancer vs. no cancer or non-skin cancer vs. no cancer.

CAV, cardiac allograft vasculopathy; NF-MACE, non-fatal major adverse cardiac events, defined as the development of myocardial infarction, new congestive heart failure, need for percutaneous coronary intervention/angioplasty, pacemaker or implantable cardioverter-defibrillator placement, and stroke; ATR, any treated rejection; ACR, acute cellular rejection; AMR, antibody-mediated rejection.

### Predictors of *de novo* post-transplant malignancies

**Figure 1:**
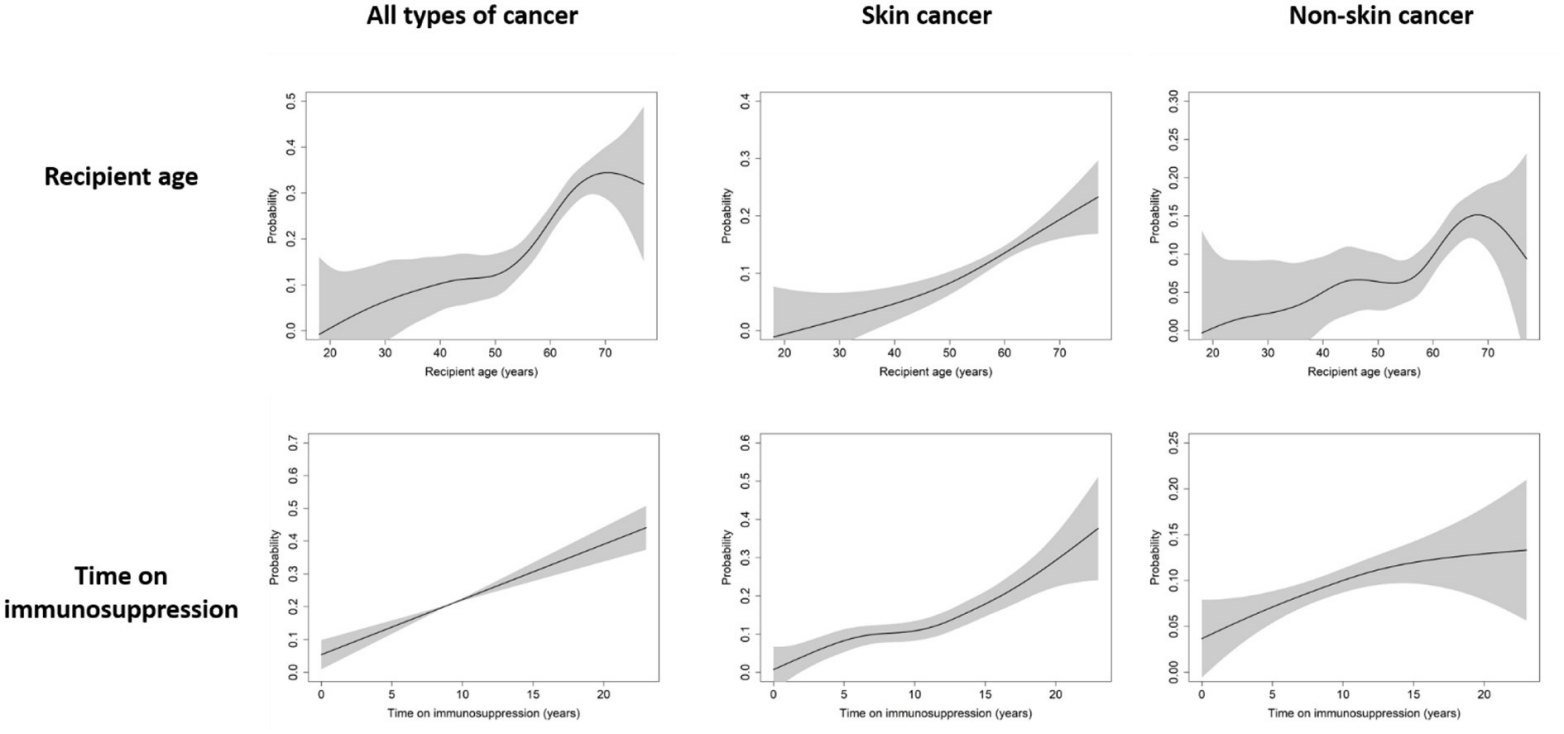
Cubic spline curve of probability for all types of cancer, skin cancer, and non-skin cancer by recipient age and time receiving immunosuppressive therapy. Black lines and gray shadows represent the estimated probability and the 95% confidence intervals for post-transplant malignancy development, respectively.

Next, we evaluated the predictors of *de novo* PTMs using univariable and multivariable logistic regression analysis (Table 4). Older age, white race, and a longer time receiving immunosuppressive therapy were independent risk factors for all types of *de novo* malignancies. However, the predictors of skin cancer and non-skin cancer were different. Although recipient age and time receiving immunosuppressive therapy were common independent predictors, white ethnicity and male sex were independently associated only with skin cancer, not with non-skin cancer. Cubic spline curves of probability for all types of cancer, skin cancer, and non-skin cancer by recipient age and time receiving immunosuppressive therapy are shown in Figure 1.

**Table 4:** Univariable and multivariable logistic regression analysis for post-transplant malignancy development.

**Variables**	**All types of cancer**			
	**Univariable**		**Multivariable**	
	**OR (95% CI)**	***P***	**OR (95% CI)**	***P***
**Age**	1.06 (1.05–1.08)	<0.001	1.07 (1.05–1.09)	<0.001
**Time on IS**	1.10 (1.07–1.14)	<0.001	1.13 (1.09–1.17)	<0.001
**White race**	3.00 (1.95–4.62)	<0.001	2.15 (1.35–3.42)	0.001
**Male**	1.62 (1.10–2.39)	0.015	1.42 (0.92–2.20)	0.115
**Ischemic time**	1.00 (1.00–1.01)	0.025	1.00 (1.00–1.00)	0.266
**Pre-HTx MCS**	0.44 (0.27–0.73)	0.001	0.73 (0.43–1.26)	0.261
	**Skin cancer**			
	**Univariable**		**Multivariable**	
	**OR (95% CI)**	***P***	**OR (95% CI)**	***P***
**Age**	1.07 (1.04–1.09)	<0.001	1.07 (1.04–1.09)	<0.001
**Time on IS**	1.12 (1.07–1.16)	<0.001	1.15 (1.10–1.21)	<0.001
**White race**	7.99 (3.47–18.40)	<0.001	5.22 (2.22–12.26)	<0.001
**Male**	2.18 (1.26–3.78)	0.005	2.00 (1.08–3.71)	0.028
**Ischemic time**	1.00 (1.00–1.01)	0.017	1.00 (1.00–1.01)	0.238
**Pre-HTx MCS**	0.56 (0.31–1.03)	0.061	1.06 (0.54–2.09)	0.870
	**Non-skin cancer**			
	**Univariable**		**Multivariable**	
	**OR (95% CI)**	***P***	**OR (95% CI)**	***P***
**Age**	1.05 (1.02–1.07)	<0.001	1.05 (1.02–1.07)	<0.001
**Time on IS**	1.06 (1.01–1.10)	0.008	1.06 (1.02–1.11)	0.006
**White race**	1.27 (0.76–2.11)	0.365	1.00 (0.58–1.71)	0.989
**Male**	1.07 (0.64–1.79)	0.793	0.94 (0.55–1.61)	0.821
**Ischemic time**	1.00 (1.00–1.00)	0.598	1.00 (1.00–1.00)	0.814
**Pre-HTx MCS**	0.37 (0.17–0.82)	0.014	0.53 (0.24–1.20)	0.127

OR, odds ratio; CI, confidence interval; IS, immunosuppression; Pre-HTx MCS, pre-transplant mechanical circulatory support.

Regarding the recipient age, all types of cancer showed a steep increase in probability from the ages of 50–70, and the probability of developing non-skin cancer also tended to increase steeply from the ages of 55–68. For skin cancer, the probability continued to increase in proportion to recipient age. For immunosuppressive therapy, the longer a patient received immunosuppressive therapy, the greater the risk for any type of cancer.

## Discussion

PTM is a significant cause of long-term morbidity and mortality in HTx recipients (2–5). However, the incidence, characteristics, long-term outcomes, and predictors of *de novo* PTM have not been well-established. In this study, 20.8% of patients developed *de novo* PTMs (241 cancers) during a median follow-up period of 11.5 years, which is consistent with the recent ISHLT registry data (8). Skin cancers were the most frequent types of malignancy (highest in white patients), followed by prostate, lung, and breast cancers. At the time of initial diagnosis of PTM, 42.3% of cancers had multiple or extensive disease status. Although most cancers (88.8%) were surgically resected at initial presentation, about half (47.3%) recurred or progressed. Similar to a previous study by Yagdi et al. patients with PTM had a significantly higher mortality than those without PTM (13). Patients without PTM had a significantly higher overall survival than those with skin cancer and non-skin cancer. Older age, white race, and a longer time receiving immunosuppressive therapy were independent risk factors for all types of *de novo* malignancies.

Survival following HTx has improved significantly with the advent of better immunosuppressive therapy (8, 9). However, excessive immunosuppression is known to increase the risk of serious infections, renal dysfunction, and PTM development in the long term (8, 9). Therefore, balancing immunosuppression to prevent rejection while minimizing infection and PTM development is a major challenge in HTx. Chronic immune suppression, and recipient's risk factors including age and genetic predisposition to cancer play important roles in PTM (1, 2, 8, 9, 12, 14–16), however, it is unclear which immunologic mechanisms play significant roles in the pathogenesis of PTM.

Non-melanoma skin cancers were the most frequent types of malignancy (57.3%), which is in accordance with previous studies (4, 17). This consistent finding highlights the importance of cancer screen in HTx patients, especially for the skin cancer. Recently, Bottomley et al. (18) reported that the accumulation of senescent T cells is a strong predictor of squamous cell carcinoma development and recurrence in a high-risk, long-term renal transplant recipient cohort. Similarly, in HTx recipients, senescent T cells may accumulate during chronic immunosuppression and reduce immune surveillance of tumorigenic activity, resulting in overt squamous cell carcinoma (18–22). These findings highlight the importance of cancer screen in HTx patients, especially for the skin cancer. Detailed underlying immunologic mechanisms should be studied to prevent skin cancer and other PTMs.

Regarding PTLD, a relationship between age and Ebstein-Barr virus (EBV) serostatus exists (23). According to a previous study by Higgins et al. (24), incidence of PTLD was the highest in young (<35 years) HTx patients with respect to then normal population. In our cohort, mean age at transplant of 7 HTx patients with PTLD was younger (57.4 ± 5.9) than HTx patients with other PTM (60.8 ± 9.3). Median time between HTx and PTLD was 8.5 [5.4–12.2] years and all patients were presented with late onset PTLD (>1 year HTx). All 7 patients with PTLD underwent surgical resection, however, 5 (57.1%) experienced recurrence and 6 (85.7%) died during follow-up. In our cohort, any significance of serologic EBV mismatch between donor and recipient could not be analyzed due to limited data availability. Considering poor prognosis of PTLD in HTx patients, larger, multicenter studies to assess the surveillance strategies focusing on EBV monitoring and prophylactic treatment approaches are needed.

In our cohort, patients with skin cancers had a significantly increased incidence of CAV and a lower 10-year freedom from CAV than patients without PTM. CAV is considered to be a result from metabolic and immune medicated injury and PTM is considered to be associated with over immunosuppression. Therefore, higher incidence of CAV in HTx recipients with skin cancer is more likely due to shared risk factor, such as old age and longer time of immunosuppression after HTx, rather than due to common pathogenic mechanisms.

CNI is known to promote cancer progression (25, 26), however, mTOR inhibitors have potential benefits in decreasing the PTM burden in terms of virus-associated malignancies and anticancer properties (27). Clinical benefits of mTOR inhibitors for PTM have been reported in kidney transplant recipients (28) and, more recently, in HTx recipients (3). Rivinius et al. (29). analyzed the distribution of malignancies in HTx patients from a single center and evaluated the risk factors. They reported significantly lower recurrences of cutaneous malignancy with mTOR inhibitor after the initial diagnosis of tumor as well as lower non-skin cancer. However, a 5-year study that included 78 HTx recipients randomized to receive tacrolimus and mycophenolate mofetil (MMF), tacrolimus and SRL, or SRL and MMF, found no difference in cancer incidence among the three groups (30). The Scandinavian Heart Transplant Everolimus De Novo Study with Early Calcineurin Inhibitor Avoidance trial (31) showed that everolimus was associated with a significant improvement in renal function and a significant reduction in CAV for *de novo* HTx recipients compared to cyclosporine. However, there was significantly more acute rejection, potentially counteracting the aforementioned benefits (1, 31). Therefore, maintenance immunosuppression regimens should be personalized after multidisciplinary discussion, considering the risks and benefits for each patient.

Older age and extended time receiving immunosuppressive therapies were common independent risk factors for all types of cancer and were associated with increased mortality. Enhanced cancer screening and individualized immunosuppressive therapy in these high-risk patients may improve their outcomes (32). The ISHLT guidelines recommend that HTx recipients undergo close skin cancer surveillance, including education on preventive measures and yearly dermatologic examinations. Recommendations regarding screening for prostate, lung, and breast cancer in the general population should also be followed in HTx recipients (33). Additionally, it is recommended that chronic immunosuppression be minimized where possible, particularly in patients at high risk for malignancy (33). Considering the increased burden of *de novo* PTM in HTx recipients, additional effort needs to be directed toward formulating evidence-based cancer screening recommendations and optimized immunosuppressive therapy protocols for these patients (2).

This study has several potential limitations. First, our study subjects could not fully represent real-world HTx recipients with *de novo* PTMs. Although the patients were consecutively enrolled, the study cannot be free from the limitations of a single-center observational study and due to retrospective nature of the study, possible risk factors in association with *de novo* PTM including detailed family history, viral serology, or smoking history were not completely evaluated. Second, detailed cancer-related information was not fully standardized due to each cancer's varied types and stages. Third, we were unable to conclude the role of specific immunosuppressive agents on the development of PTMs due to diverse immunosuppression regimens and their changes during the follow-up period. Initial immunosuppressive regimens after HTx were similar between two groups (Supplementary Table 2). The percentages of patients who had switched to mTOR inhibitors during follow up were also similar between two groups, however, we could not assess whether switching was made at the time of diagnosis of malignancies in PTM group.

In conclusion, older age, white race, and extended time receiving immunosuppressive therapy were identified as independent risk factors for PTM associated with increased mortality in this study. Further research is necessary for the prevention and early detection of PTM in HTx recipients.

## Data availability statement

Relevant data are available from the corresponding author on reasonable request.

## Ethics statement

The studies involving human participants were reviewed and approved by the Cedars-Sinai Institutional Review Board. The patients/participants provided their written informed consent to participate in this study.

## Author contributions

J-CY, I-CK, J-OC, E-SJ, KN, EK, DC, MK, JP, DR, FE, and JK contributed to conception and design of the study. J-CY organized the database. J-CY, DK, and HL performed the statistical analysis. J-CY and DK wrote the first draft of the manuscript. All authors contributed to manuscript revision, read, and approved the submitted version.

## Funding

This work was supported by the National Research Foundation of Korea (NRF) grant funded by the Ministry of Science and ICT (NRF-2021R1F1A1063430), by the Catholic Medical Center Research Foundation (2022), and by a grant from the Korean Society for Transplantation. The funders had no role in study design, data collection and analysis, decision to publish, or preparation of the manuscript.

## Conflict of interest

DC received research grants from Amgen, Biocardia, and Mesoblast and has moderate stock interest in Abbot Laboratories, Abbvie Inc, Repligen Corporation, Amarin Corporation, and Portola Pharmaceuticals. JP received research grants from Alexion Pharmaceuticals, Pfizer, Alnylam Pharmaceuticals, and Astra Zeneca. DR received research grants from Abiomed, Cardiac Assist, Inc., and Thoratec LLC, consultancy fees from Abbot Laboratories and Baxter Healthcare, and non-financial support from Medtronic Vascular. FE received a research grant from TransMedics Inc. JK received research grants from CareDx Inc., Sanofi-Genzyme, and CSL-Behringer.

The remaining authors declare that the research was conducted in the absence of any commercial or financial relationships that could be construed as a potential conflict of interest.

## Publisher's note

All claims expressed in this article are solely those of the authors and do not necessarily represent those of their affiliated organizations, or those of the publisher, the editors and the reviewers. Any product that may be evaluated in this article, or claim that may be made by its manufacturer, is not guaranteed or endorsed by the publisher.
