# Supplementary material for: Characteristics, outcomes, and predictors of de novo malignancy after heart transplantation
Source: Front Cardiovasc Med. 2022 Aug 8;9:939275. doi: 10.3389/fcvm.2022.939275 (PMC9393331; doi:10.3389/fcvm.2022.939275)
Supplement: Supplementary file 1 [file Table_1.docx]

**Supplementary table 1. Clinical outcome of patients with and without post-transplant malignancy.**

| **Variables** | **Non-melanoma skin cancer (n=108)** | | **Melanoma**  **(n=8)** | **Non-skin cancer**  **(n = 90)** | **No cancer**  **(n = 783)** | | ***P*** | |
| --- | --- | --- | --- | --- | --- | --- | --- | --- |
| **Mortality outcomes** |  | |  |  |  | |  | |
| Deaths | 45 (41.7%) | | 4 (50.0%) | 59 (65.6%)^*^ | 305 (39.0%) | | < 0.001 | |
| Cause of death |  |  | | | | < 0.001 | |  |
| - Cardiac | 6 (13.3%) | | 0 (0%) | 3 (5.1%) | 132 (43.3%) | |  | |
| - Infection | 8 (17.8%) | | 0 (0%) | 15 (25.4%) | 66 (21.6%) | |  | |
| - Malignancy | 11 (24.4%) | | 4 (100%) | 33 (55.9%) | 0 (0%) | |  | |
| - Renal | 3 (6.7%) | | 0 (0%) | 1 (1.7%) | 14 (4.6%) | |  | |
| - Cerebrovascular | 0 (0%) | | 0 (0%) | 2 (3.4%) | 10 (3.3%) | |  | |
| - Others | 17 (37.8%) | | 0 (0%) | 5 (8.5%) | 83 (27.2%) | |  | |
| **Morbidity outcomes** |  | |  |  |  | |  | |
| CAV | 43 (39.8%)* | | 3 (37.5%) | 26 (28.9%) | 183 (23.4%) | | 0.002 | |
| NF-MACE | 33 (30.6%) | | 4 (50.0%) | 30 (33.3%) | 199 (25.4%) | | 0.128 | |
| ATR | 21 (19.4%) | | 2 (25.0%) | 13 (14.4%) | 145 (18.5%) | | 0.740 | |
| ACR | 12 (11.1%) | | 1 (12.5%) | 6 (6.7%) | 74 (9.5%) | | 0.740 | |
| AMR | 6 (5.6%) | | 0 (0%) | 8 (8.9%) | 56 (7.2%) | | 0.695 | |
| 10-year freedom from CAV | 65.7%* | | 62.5% | 74.4% | 78.5% | | 0.020 | |
| 10-year freedom from NF-MACE | 74.1% | | 50.0% | 69.4% | 76.7% | | 0.219 | |
| 10-year freedom from ATR | 80.6% | | 75.0% | 86.1% | 80.9% | | 0.509 | |
| 10-year freedom from ACR | 88.9% | | 87.5% | 94.4% | 90.6% | | 0.739 | |
| 10-year freedom from AMR | 94.4% | | 100% | 93.1% | 92.4% | | 0.802 | |

CAV = cardiac allograft vasculopathy; NF-MACE = non-fatal major adverse cardiac events, defined as the development of myocardial infarction, new congestive heart failure, need for percutaneous coronary intervention/angioplasty, pacemaker or implantable cardioverter-defibrillator placement, and stroke; ATR = any treated rejection; ACR = acute cellular rejection; AMR = antibody-mediated rejection.

*Significant with a P-value < 0.05 for non-melanoma skin cancer vs. no cancer, melanoma vs. no cancer or non-skin cancer vs. no cancer

**Supplementary Table 2. Immunosuppressive regimens at the time of discharge after HTx.**

| **Variables** | **PTM (n = 206)** | **No PTM (n = 783)** | ***P*** |
| --- | --- | --- | --- |
|  |  |  |  |
| Immunosuppressive regimens |  |  |  |
| - CNI + azathioprine | 13 (6.3%) | 53 (6.8%) | 0.877 |
| - CNI + MPA | 176 (85.4%) | 651 (78.7%) | 0.525 |
| - CNI + mTORi | 4 (1.9%) | 30 (3.8%) | 0.281 |

CNI, Calcineurin inhibitors; MPA, Mycophenolic acid; mTORi, mammalian target of rapamycin inhibitor
